# Genome Sequences of *Campylobacter jejuni* 81-176 Variants with Enhanced Fitness Relative to the Parental Strain in the Chicken Gastrointestinal Tract

**DOI:** 10.1128/genomeA.00006-14

**Published:** 2014-02-06

**Authors:** Jeremiah G. Johnson, Samuel Carpentier, Rachel R. Spurbeck, Sukhinder K. Sandhu, Victor J. DiRita

**Affiliations:** a Department of Microbiology and Immunology, University of Michigan Medical School, Ann Arbor, Michigan, USA; b Swift Biosciences, Inc., Ann Arbor, Michigan, USA

## Abstract

*Campylobacter jejuni* is a major cause of food-borne infections in the United States due to its ability to asymptomatically colonize the gastrointestinal tracts of chickens. Using competition assays with parental *C. jejuni* 81-176, variants with consistently improved fitness in chicken ceca relative to the parental strain were identified and sequenced.

## GENOME ANNOUNCEMENT

*Campylobacter jejuni* is a leading cause of bacterium-derived food-borne infections in the United States, with human infection often resulting in moderate to severe bloody diarrhea (1). The prevalence of infection is primarily due to its ability to asymptomatically colonize the gastrointestinal tracts of agriculturally relevant animals, especially chickens (2). At harvest, bacteria are often released from the gastrointestinal tract, contaminating the meat products as a result. Much work has centered on the identification and characterization of factors that allow the organism to colonize the chicken gastrointestinal tract with the aim of developing anti-*Campylobacter* strategies for the food industry (3).

To identify the fitness factors of *C. jejuni*, White Leghorn day-of-hatch chickens (Charles River Laboratories, Wilmington, MA) were colonized with equal numbers (10^5^ CFU) of parental *C. jejuni* 81-176 and an otherwise isogenic streptomycin-resistant derivative, *C. jejuni* DRH212 (4). After 7 days, the cecal contents were collected and plated on *Campylobacter*-selective medium containing 10 µg/ml trimethoprim and grown at 37°C under microaerobic conditions (85% N_2_, 10% CO_2_, 5% O_2_). The recovered isolates were replica-plated onto medium containing 10 µg/ml trimethoprim and 2 mg/ml streptomycin for the determination of strain ratios (81-176:DRH212) within the cecal samples. In several chickens, variants of *C. jejuni* DRH212 became predominant, outcompeting parental *C. jejuni* 81-176 by orders of magnitude. Two of these variants, *C. jejuni* UMCW7 and UMCW9, were isolated and confirmed in subsequent colonization studies to reproducibly outcompete parental *C. jejuni* 81-176 *in vivo*.

Genomic DNA was extracted from DRH212, UMCW7, and UMCW9 using a Qiagen blood and tissue kit (Qiagen, Valencia, CA). Approximately 1 µg of genomic DNA (gDNA) was fragmented to an average size of 200 bp using a Covaris M220 (Covaris, Woburn, MA) and prepared for next-generation sequencing using an Accel-NGS kit (Swift Biosciences, Inc., Ann Arbor, MI). Genomic fragments were sequenced using an Ion Torrent PGM sequencer and Ion 316 Chip (Life Technologies, Carlsbad, CA). Using 500 flows that were formulated for 200-nucleotide chemistry, we obtained 169- to 181-nucleotide read lengths with 2.9 to 3.0 million reads per genome. High-quality reads (>q20) were trimmed to 200 bp using the FastX-Toolkit (Hannon Lab). These reads were processed using either NextGENe (SoftGenetics, State College, PA) for DRH212 or MIRA4 (5) for UMCW7 and UMCW9 to yield *de novo* assemblies. The resulting assemblies yielded 24 contigs for DRH212 (average total coverage, 133×; N_50_, 97 kb; total length, 1.41 Mb), 33 contigs for UMCW7 (average total coverage, 133×; N_50_, 101 kb; total length, 1.71 Mb), and 38 contigs for UMCW9 (average total coverage, 217×; N_50_, 112 kb; total length, 1.66 Mb). A BLAST search of the assembled contigs confirmed that each strain carried one chromosome and one plasmid that exhibits high similarity to *C. jejuni* 81-176 pVir.

### Nucleotide sequence accession numbers.

This whole-genome shotgun project has been deposited in DDBJ/EMBL/GenBank under accession no. AZNT00000000, AZNS00000000, and AZQV00000000 for strains DRH212, UMCW7, and UMCW9, respectively. The versions described in this paper are the first versions, AZNT01000000, AZNS01000000, and AZQV01000000 for DRH212, UMCW7, and UMCW9, respectively.
